# Simulated microgravity inhibits cell focal adhesions leading to reduced melanoma cell proliferation and metastasis *via* FAK/RhoA-regulated mTORC1 and AMPK pathways

**DOI:** 10.1038/s41598-018-20459-1

**Published:** 2018-02-28

**Authors:** Xin Tan, Aizhang Xu, Tuo Zhao, Qin Zhao, Jun Zhang, Cuihong Fan, Yulin Deng, Andrew Freywald, Harald Genth, Jim Xiang

**Affiliations:** 1 Aerospace Institute of Medical Engineering and Biotechnology, School of Life Sciences, Beijing Institute of Technology, Beijing, 10081 China; 2 Cancer Research, Saskatchewan Cancer Agency, Saskatoon, Saskatchewan S7N 4H4 Canada; 3 Department of Oncology, University of Saskatchewan, Saskatoon, Saskatchewan S7N 5E5 Canada; 4 Department of Pathology, University of Saskatchewan, Saskatoon, Saskatchewan S7N 5E5 Canada; 5 Hannover Medical School, D-30625 Hannover, Germany

## Abstract

Simulated microgravity (SMG) was reported to affect tumor cell proliferation and metastasis. However, the underlying mechanism is elusive. In this study, we demonstrate that clinostat-modelled SMG reduces BL6-10 melanoma cell proliferation, adhesion and invasiveness *in vitro* and decreases tumor lung metastasis *in vivo*. It down-regulates metastasis-related integrin α6β4, MMP9 and Met72 molecules. SMG significantly reduces formation of focal adhesions and activation of focal adhesion kinase (FAK) and Rho family proteins (RhoA, Rac1 and Cdc42) and of mTORC1 kinase, but activates AMPK and ULK1 kinases. We demonstrate that SMG inhibits NADH induction and glycolysis, but induces mitochondrial biogenesis. Interestingly, administration of a RhoA activator, the cytotoxic necrotizing factor-1 (CNF1) effectively converts SMG-triggered alterations and effects on mitochondria biogenesis or glycolysis. CNF1 also converts the SMG-altered cell proliferation and tumor metastasis. In contrast, mTORC inhibitor, rapamycin, produces opposite responses and mimics SMG-induced effects in cells at normal gravity. Taken together, our observations indicate that SMG inhibits focal adhesions, leading to inhibition of signaling FAK and RhoA, and the mTORC1 pathway, which results in activation of the AMPK pathway and reduced melanoma cell proliferation and metastasis. Overall, our findings shed a new light on effects of microgravity on cell biology and human health.

## Introduction

The cytoskeleton is a cellular structural scaffold that determines cell shape, provides an intracellular transport system, drives cell migration and actively controls cell survival and proliferation^1^. The cytoskeleton of eukaryotic cells is composed of three basic types of filaments (actin filaments, microtubules and intermediate filaments). The extracellular matrix, integrin receptors and cytoskeleton interact at sites called focal adhesions^2^. The integrin-binding proteins paxillin, vinculin and talin recruit focal adhesion kinase (FAK) to focal adhesions composed of dynamic groups of structural and catalytic proteins, that transduces external integrin-mediated signals into cells, leading to the activation of multiple cytoplasmic signaling molecules, including small GTPases^3^.

The ras homolog gene-family member (Rho) GTPases are important components of the signaling network represented by RhoA, ras-related C3 botulinum-toxin substrate-1 (Rac1) and cell division-control protein-42 (Cdc42) molecules, that regulate activities of actin-binding proteins to control actin crosslinking and stress fiber formation. This allow Rho family GTPases to regulate cytoskeleton-mediated cell shape, motility and division^4^. Rho family members also control multiple intracellular signaling pathways^5–9^, including signaling initiated by the mammalian target of rapamycin complex-1 (mTORC1)^5,6,10^.

The mTORC1 kinase and another evolutionary conserved signaling molecule, the AMP-activated protein kinase (AMPK), have important functions in the regulation of cellular metabolism for maintenance of energy homeostasis^11^. mTORC1 is a serine/threonine protein kinase, which functions as a central regulator of cell proliferation and growth through the activation of the S6 kinase (S6K), and the eukaryotic initiation factor 4E (EIF4E)^12^. It acts as a sensor of cellular energy status and triggers glycolysis utilization by activation of hypoxia-inducible factor-1α transcription factor^13^. AMPK also acts as a sensor of cellular energy status and activates mitochondrial biogenesis and fatty acid oxidation for energy production *via* activation of the Une-51-like kinase-1 (ULK1)^14^.

The simulated microgravity (SMG) has been reported to alter cytoskeleton and extracellular matrix proteins^15–17^. SMG was also found to affect tumor cell adhesion, proliferation, aggressiveness and metastasis^16,18–20^, and to induce cell autophagy^21,22^. Recently, SMG has been demonstrated to inhibit the mTORC1 pathway^22,23^. However, the molecular mechanism underlying the above SMG-induced changes in cell biology and cellular pathways is still elusive. We previously established a three-dimensional clinostat modeling SMG environment to investigate molecular mechanisms regulating SMG-induced cellular apoptosis, and found that SMG promotes apoptosis of B16 melanoma BL6-10 cells by suppressing NF-κB-mediated anti-apoptotic events and by inhibiting DNA-damage response pathways^24^. We have recently discovered that SMG reduced formation of cellular focal adhesions, which was associated with the SMG-induced down-regulation of Rho family proteins^25^.

In this study, we further investigated SMG effects on BL6-10 melanoma cell proliferation, invasiveness and metastasis by using the clinostat-modelled SMG^24^. More importantly, we also analyzed the potential molecular mechanism regulating the SMG-induced cellular responses by monitoring cell focal adhesions and associated signaling molecules, such as the FAK kinase and Rho family proteins (RhoA, Rac1 and Cdc42), as well as molecules involved in the FAK/RhoA-controlled mTORC1 pathway-related molecules (AKT, S6K, EIF4E) and AMPK^12–14^ in cells under SMG. We found that SMG reduced formation of cell focal adhesions, leading to decreased melanoma cell growth and metastasis. This was achieved through the FAK/RhoA-mediated inhibition of the mTORC1 pathway and the FAK/RhoA-induced activation of the AMPK pathway.

## Results

### Simulated microgravity inhibits both proliferation of melanoma cells and their metastatic activity

**Figure 1:**
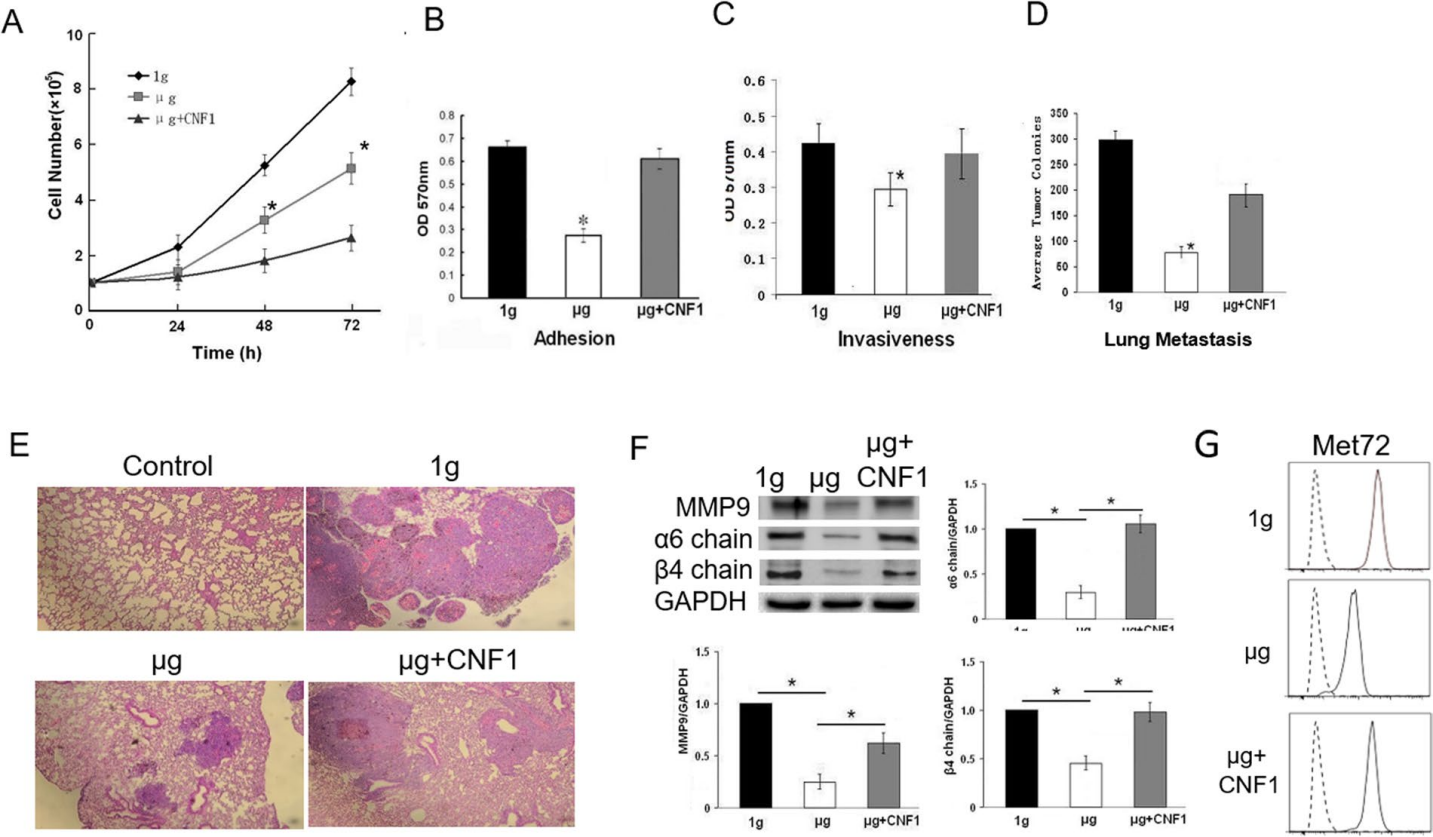
Simulated microgravity inhibits BL6-10 tumor cell proliferation and metastasis. (**A**) BL6-10 tumor cells were cultured in flasks under normal gravity (1 g) or cultured with or without CNF1 under SMG (µg + CNF1 or µg). Cells under 1 g, µg and µg + CNF1 were counted daily for three days to quantify cell proliferation. (**B,C**) BL6-10 tumor cells cultured in chamber slides under 1 g, µg and µg + CNF1 were subjected to cell adhesion and invasion assays using CytoSelect™ 24-Well Cell Adhesion Assay kit (**B**) and CytoSelect™ 24-Well Cell Invasion Assay kit (**C**). (**D,E**) BL6-10 cells subjected to 1 g, µg and µg + CNF1 were i.v. injected into C57BL/6 mice. Mouse lungs were collected 21 days after injection, and black tumor lung colonies were counted (**D**) and confirmed by histological examination of lung tissue sections with H.E staining (**E**). (**F**) Lysates prepared from BL6-10 cells grown at 1 g, µg and µg + CNF1 for 3 days were subjected to SDS-PAGE. Proteins were transferred onto PVDF membranes, blotted with the indicated antibodies. Western blot band signals were quantified by chemiluminescence. Densitometric values were normalized to matching GAPDH controls. Data represent the mean ± SD of three independent experiments. (**G**) BL6-10 tumor cells grown at 1 g, µg and µg + CNF1 for 3 days were stained with anti-Met72 antibody (solid lines) or isotype-matched control antibody (dotted lines), followed by flow cytometry analysis. *p < 0.05 versus 1 g and µg + CNF1 groups. One representative experiment of two is shown.

To assess the effect of SMG on cell growth, we performed a cell proliferation assay, and found that *in vitro* growth of BL6-10 cells was greatly inhibited under SMG (µg) compared to cells under normal gravity (1 g) (Fig. 1A). Our cell adhesion assay also revealed that adhesion of BL6-10 cells was significantly reduced under SMG in comparison to cells maintained under 1 g (Fig. 1B). To analyze the ability of melanoma cells to degrade and invade surrounding extracellular matrix, we performed an invasion assay using Boyden chambers pre-coated with basement membrane components provided with the CytoSelect™ 24-Well Cell Adhesion Assay kit. We found that invasiveness of BL6-10 tumor cells under SMG conditions was significantly reduced compared to control BL6-10 tumor cells analyzed at normal gravity (Fig. 1C). To assess the effect of SMG on the metastatic activity, we i.v. injected the highly lung metastatic BL6-10 cells grown under 1 g or SMG condition into C57BL/6 mice, and quantified mouse lung tumor colonies in lungs 21 days later. This experiment demonstrated that numbers of metastatic BL6-10 melanoma lung colonies were significantly reduced in mice injected with BL6-10 cells grown under SMG, compared to their numbers in mice injected with BL6-10 cells that were grown under 1 g condition (Fig. 1D). In addition, sizes of metastatic colonies in mice injected with BL6-10 cells subjected to SMG were much smaller than those in mice injected with control BL6-10 cells (Fig. 1E). Overall, these data indicate that SMG inhibits aggressiveness of melanoma cells.

### Simulated microgravity inhibits expression of metastasis-related molecules

Previous reports demonstrated that integrin α6β4 and matrix metalloproteinase-9 (MMP9) directly affected tumor cell metastasis^26,27^, and expression of BL6-10 melanoma cell-surface 72 Kd-glycoprotein, Met72, was associated with high tumor metastasis to lungs^28^. We therefore performed Western blotting and flow cytometry analyses to assess expression of MMP9 and integrin α6β4, and the presence of Met72 on the cell surface. Interestingly, we found that the pro-metastatic MMP9 and the integrin α6β4 protein as well as the metastasis-associated Met72 were both down-regulated in melanoma cells under SMG (Fig. 1F,G), indicating that SMG most likely inhibits melanoma cell invasiveness and metastasis by suppressing expression of these metastasis-related molecules.

### Simulated microgravity alters cytoskeleton organization and dramatically reduces formation of focal adhesions

**Figure 2:**
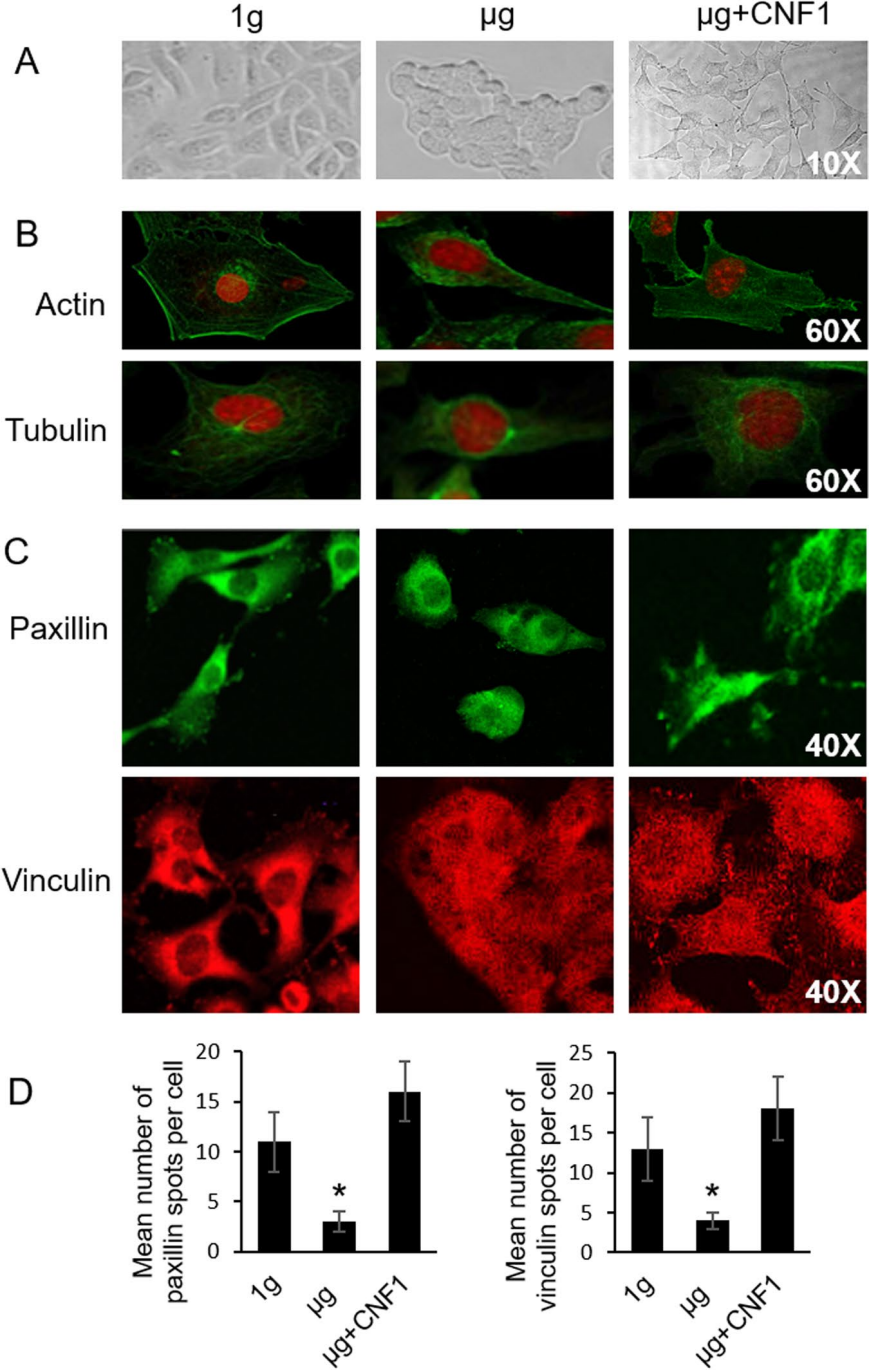
Simulated microgravity alters cytoskeleton and inhibits focal adhesions. BL6-10 cells were cultured in chamber slides for 1 day at 1 g, µg and µg + CNF1, and cells were analyzed by light microscopy (**A**). The cells were also stained with FITC-phalloidin (green) plus PI (red) and FITC-anti-tubulin antibody (green) plus PI (red), respectively (**B**) or stained with anti-paxillin (green) and anti-vinculin (red) antibody (**C**) and analyzed by fluorescence microscopy. (**D**) Paxillin and vinculin spots were counted for each cell. Approximately, 20 cells were analyzed per experimental condition. *p < 0.05 versus indicated groups. One representative experiment of two is shown.

BL6-10 cells growing on the surface of culture chamber slides under 1 g condition displayed flat and irregular morphology, while under SMG, they remained attachment to the bottom of culture chamber slides, and acquired a cobblestone-like morphology and aggregated into clusters under SMG (Fig. 2A), mimicking the behaviour typical for non-invasive epithelial-like cells^29^. This behaviour also indicated that their cytoskeleton structures may have been changed. To assess cytoskeleton alteration triggered by SMG, we stained cells with fluorescein isothiocyanate (FITC)-labeled phalloidin and FITC-labeled anti-α-tubulin antibodies, which allowed us to monitor status of microfilaments and microtubules, respectively. Control cells cultured under 1 g spread out evenly over the substrate and displayed abundant lamellipodia (membrane ruffles at the leading edge), stress fibres (actin/myosin bundles) and filopodia (membrane protrusion)^4^, while cells exposed to SMG showed a dramatic decrease in lamellipodia, stress fibres and filopodia (Fig. 2B). These data were consistent with our previous report^24^, and indicate that SMG alters cytoskeleton structure. Since integrin-binding proteins paxillin and vinculin, which are involved in recruiting FAK to focal adhesions, are integral components of these structures^3^, we stained cells under SMG with anti-paxillin or vinculin antibodies, and analyzed them by fluorescence microscopy to assess formation of cell focal adhesions. We found that focal adhesions (means of paxillin or vinculin spots per cell)^30^ were substantially reduced in cells under SMG in comparison with control cells under 1 g condition (Fig. 2C,D), indicating that SMG not only affects cell morphology and cytoskeleton, but also dramatically reduces formation of cellular focal adhesions.

### Simulated microgravity inhibits FAK and RhoA activation

**Figure 3:**
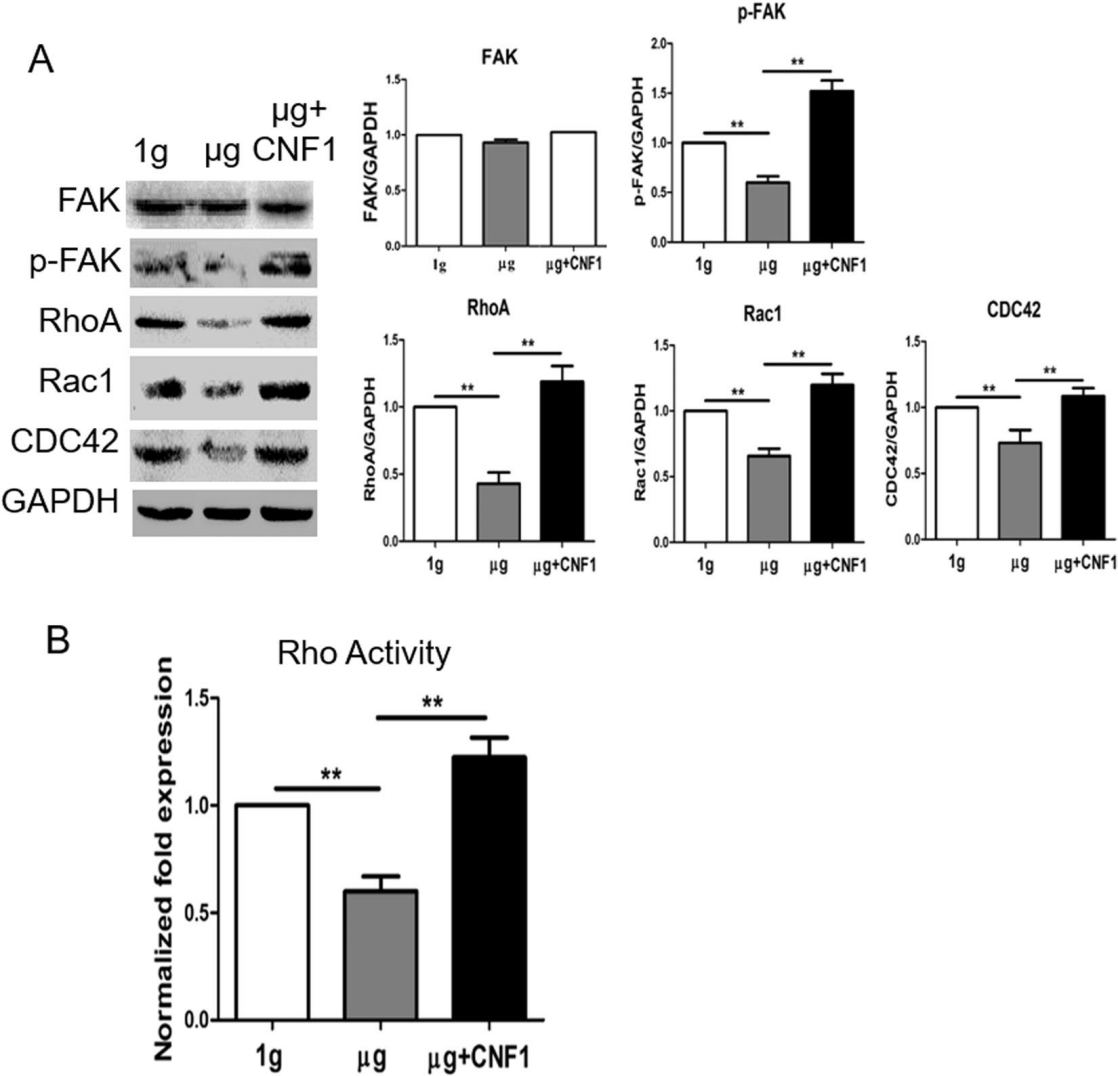
Simulated microgravity inhibits FAK and RhoA activation. (**A**) Lysates prepared from BL6-10 cells cultured for 3 days at 1 g or µg or µg + CNF1 were subjected to SDS-PAGE analysis. Proteins were transferred onto PVDF membranes and blotted with indicated antibodies. Western blot band signals were quantified by chemiluminescence. Densitometric values were normalized to matching GAPDH controls. Data represent the mean ± SD of three independent experiments. (**B**) BL6-10 cells exposed for 3 days to 1 g, µg and µg + CNF1 were analyzed for RhoA activity by using the G-LISA RhoA Activation Assay Biochem kit. Data represent the mean ± SD of three independent experiments. **p < 0.05 versus indicated groups. One representative experiment of two is shown.

To assess the effect of SMG on FAK, we performed Western blotting analysis using cell lysates derived from adherent cells in flasks positioned under SMG or 1 g condition and anti-FAK and anti-pFAK (Y397) antibodies. These experiments showed that active form of FAK, represented by FAK phosphorylated at the tyrosine residue 397 (Y397), was significantly less abundant in cells under SMG, though overall FAK expression was maintained at the same level in cells under normal 1 g condition (Fig. 3A). To assess if SMG affects expression of Rho family GTPases, we performed Western blotting analysis with anti-RhoA, anti-Rac1 and anti-Cdc42 antibodies. This revealed that SMG down-regulates expression of RhoA, Rac1 and Cdc42 (Fig. 3A). To assess the effect of SMG on RhoA activity, we performed a RhoA activity assay using G-LISA RhoA Activation Assay Biochem kit. The experiment showed that RhoA activity was significantly reduced in SMG-treated cells (Fig. 3B). Our observations thus indicate that SMG negatively regulate activities of the FAK kinase and of the RhoA GTPase.

### Simulated microgravity suppresses the mTORC1-S6K-EIF4E but activates the AMPK-ULK1 pathway

**Figure 4:**
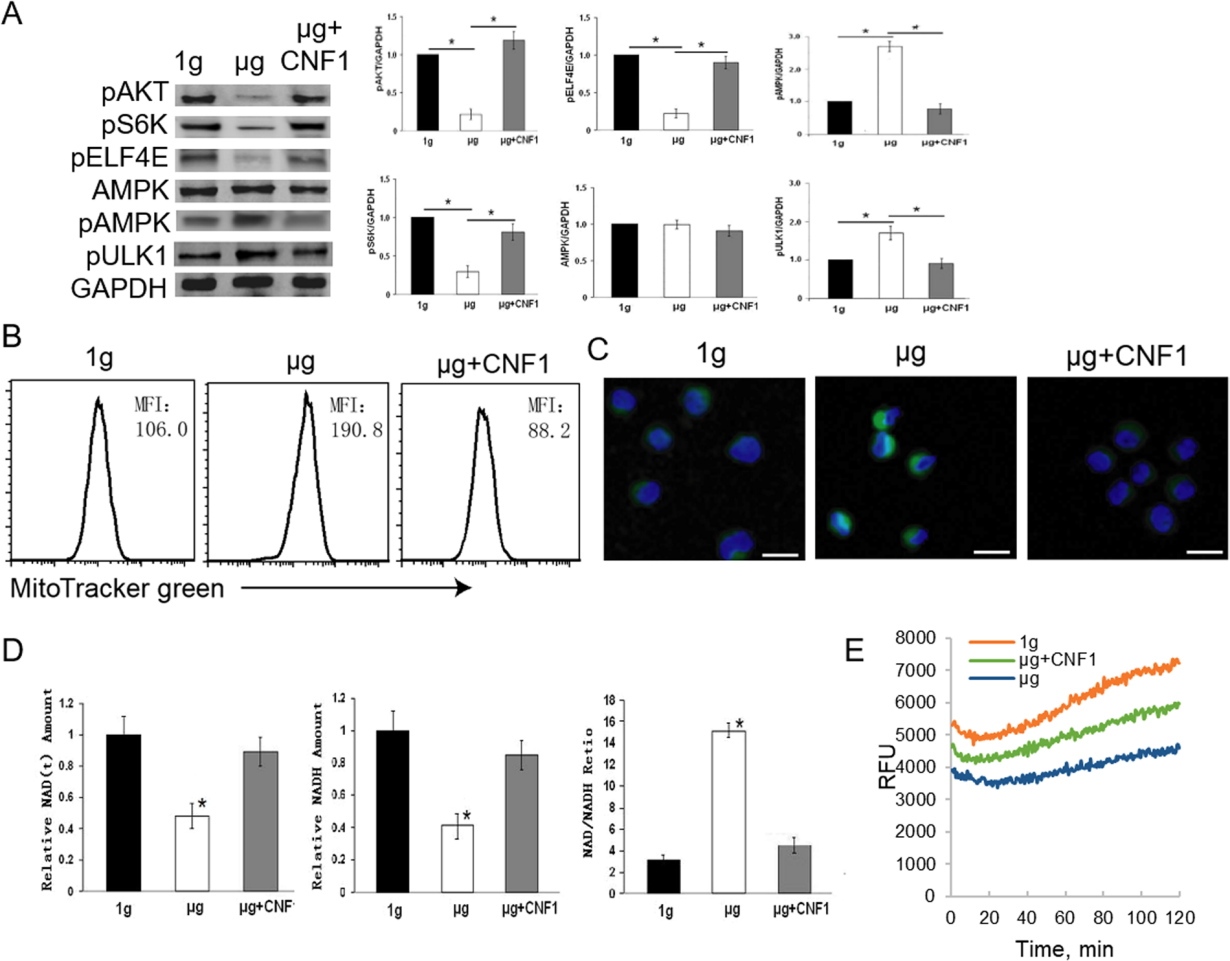
Simulated microgravity suppresses the mTORC1 but activates the AMPK pathway. (**A**) Lysates prepared from BL6-10 cells cultured for 3 days at 1 g or µg or µg + CNF1 were subjected to SDS-PAGE analysis. Proteins were transferred onto PVDF membranes and blotted with indicated antibodies. Western blot band signals were quantified by chemiluminescence. Densitometric values were normalized to matching GAPDH controls. Data represent the mean ± SD of three independent experiments. *p < 0.05 versus different groups. (**B**,**C**) BL6-10 cells cultured for 3 days at 1 g or µg or µg + CNF1 were subjected to mitochondria biogenesis assay using MiltoTracker Green kit. Cellular mitochondria biogenesis was quantified by flow cytometry (**B**). MFI: mean fluorescence intensity. Cellular mitochondria biogenesis was examined by confocal microscopy (**C**). Scale bar: 20 µm. (**D**) BL6-10 cells cultured for 3 days under 1 g or µg or µg + CNF1 were subjected to NADH assay using NAD + /NADH Quantification kit. Data represent the mean ± SD of three independent experiments. (**E**) BL6-10 cells cultured for 3 days at 1 g or µg or µg + CNF1 were subjected to cell glycolysis assay using pH-Xtra^TM^ Glycolysis Assay kit. *p < 0.05 versus indicated groups. One representative experiment of two is shown.

Since RhoA regulates the essential mTORC1 signaling pathway^5,6,10^, we investigated whether SMG affects mTORC1 pathway by assessing expression of mTORC1 up- and down-stream partners (AKT, S6K and EIF4E) in cells under SMG. Interestingly, we found that SMG reduced abundance of activated kinases pAKT (S473), pS6K (S235) and pEIF4E (S209) (Fig. 4A), indicating that SMG supresses the AKT-mTORC1-S6K-EIF4E pathway. To assess a potential effect of SMG on another conserved signaling pathway that involves AMPK, we examined phosphorylation of the AMPK and theULK1 kinases. Interestingly, we found that levels of pAMPK (T172) and pULK1 (S375) were upregulated in cells under SMG (Fig. 4A), indicating that SMG effectively activates the AMPK-ULK1 pathway.

### Simulated microgravity induces mitochondria biogenesis and reduces NADH induction

Fast growing cells use glycolysis metabolism for energy, while quiescent cells often rely on fatty acid oxidation and induce mitochondrial biogenesis to obtain energy in the form of ATP^31^. AMPK acts as a sensor of cellular energy status and is responsible for the triggering mitochondrial biogenesis and fatty acid oxidation for energy production^14^. Therefore, we attempted to assess whether SMG induces mitochondria biogenesis in cells under SMG condition. To achieve this, we stained cells with a mitochondrial dye, MitoTracker Green, and then assessed the status of mitochondria by flow cytometry and confocal microscopy. We found that compared to cells maintained under normal gravity, SMG-treated cells showed a higher mitochondrial content (Fig. 4B), and more abundant cytoplasmic mitochondria (Fig. 4C), indicating that MSG induces mitochondria biogenesis. Since NADH is produced by tricarboxylic acid cycle critical in mitochondrial oxidative phosphorylation system for production of aerobic ATP^32^, we measured NADH levels using NAD+/NADH Quantification kit to complement the above finding. We found that cells under SMG had less NAD(H) and higher ratio of NAD/NADH, compared to cells under 1 g condition (Fig. 4D), indicating that SMG inhibits NADH induction, which points towards the suppression of glycolysis metabolism. We then assessed glycolysis, and demonstrated that SMG-treated cells dramatically reduced cell glycolysis metabolism (Fig. 4E).

### CNF1 enhances activity of FAK and RhoA and restores cytoskeleton, focal adhesions, cell proliferation and metastasis in cells under SMG

Since bacterial toxin, CNF1, produced from *E. coli* cells has been found to increase focal adhesions *via* the activation of RhoA, Rac1 and Cdc42 GTPases^33,34^, we examined whether CNF1 does affect activities of FAK and RhoA, and also assessed whether NCF1 converts alterations in cytoskeleton and focal adhesions in cells under SMG. These experiments showed that CNF1 up-regulated levels of pFAK (Y397), RhoA, Rac1 and Cdc42 molecules (Fig. 3A) and enhanced RhoA activity (Fig. 3B) in cells under SMG, indicating that CNF1 enhances FAK and RhoA signaling under SMG condition. Our data also demonstrated that when cells under SMG were treated with CNF1, cytoskeleton organization and focal adhesions (means of paxillin or vinculin spots per cell)^30^ (Fig. 2A,D) and cell proliferation rates, adhesion efficiency, invasiveness and metastatic activity (Fig. 1A,E) were comparable to those characteristics of cells cultured under normal gravity. In addition, CNF1 also up-regulated expression of metastasis-related α6β4 integrin, MMP9 and Met72 in cells under SMG (Fig. 1F,G). Thus, our data indicate that CNF1 restores cytoskeleton, focal adhesions and cell proliferation and metastasis in cells under SMG *via* the activation of FAK and RhoA signaling.

### CNF1 activates mTORC1 signaling and increases NADH and glycolysis but suppresses the AMPK pathway and reduces mitochondria biogenesis in cells subjected to SMG

Since RhoA activates the mTORC1 pathway^5,6^, we then analyzed whether CNF1 affects the mTORC1 or the AMPK pathway in cells under SMG. Our experiments demonstrated that CNF1-treated cells exposed to SMG up-regulated levels of pAKT (S473), pS6K (S235) and pEIF4E (S209) while down-regulating expression of pAMPK (T172) and pULK1 (S375) by Western blotting analysis (Fig. 4A), compared to cells under SMG. In addition, our data also demonstrated that CNF1 reduced mitochondria biogenesis (Fig. 4B,C), but increased NADH induction (Fig. 4D) and glycolysis metabolism (Fig. 4E). Taken together, our data suggest that CNF1 activates the mTORC1 but suppresses the AMPK pathway in cells under SMG and mostly achieves this through the activation of FAK and RhoA signaling.

### Rapamycin inhibits the mTORC1 pathway, cell proliferation and metastasis and but activates the AMPK pathway and mitochondria biogenesis in cells under 1 g condition

**Figure 5:**
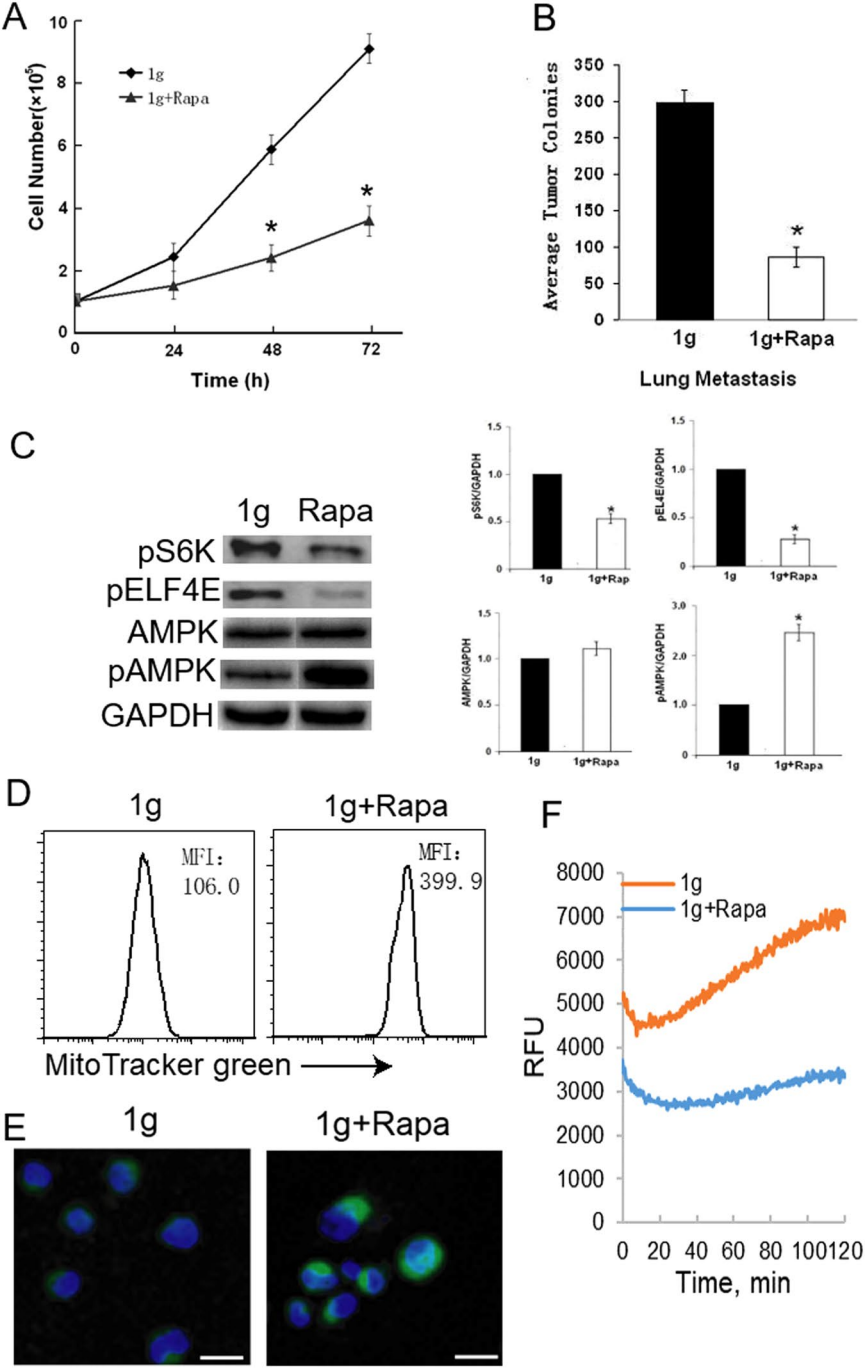
mTORC1 inhibitor rapamycin inhibits cell proliferation and metastasis, suppresses the mTORC1 pathway and activates the AMPK pathway. (**A**) BL6-10 tumor cells cultured in flasks at 1 g or 1 g + rapamycin were counted daily for three days to measure cell proliferation. (**B**) BL6-10 tumor cells cultured at 1 g and 1 g + Rapa for three days were i.v. injected into C57BL/6 mice. Mouse lungs were collected 21 days after tumor cell injection, and black tumor lung colonies were counted. (**C**) Lysates prepared from BL6-10 cells cultured for 3 days at 1 g or 1 g + rapamycin were subjected to SDS-PAGE analysis. Proteins were transferred onto PVDF membranes and blotted with indicated antibodies. Western blot band signals were quantified by chemiluminescence. Densitometric values were normalized to matching GAPDH control. Data represent the mean ± SD of three independent experiments. *p < 0.05 versus indicated groups. (**D,E**) BL6-10 cells cultured for 3 days under 1 g or 1 g + rapamycin were subjected to mitochondria biogenesis assay using MiltoTracker Green kit. Cellular mitochondria biogenesis was quantified by flow cytometry (**D**). MFI: mean fluorescence intensity. Cellular mitochondria biogenesis was measured by confocal microscopy (**E**). Scale bar: 20 µm. (**F**) BL6-10 cells cultured for 3 days under 1 g or 1 g + Rapa were subjected to cell glycolysis assay using pH-Xtra^TM^ Glycolysis Assay kit. One representative experiment of two is shown.

To assess whether SMG-induced inhibition of the mTORC1 pathway is associated with SMG-induced inhibition of cell proliferation and metastasis as well as activation of the AMPK pathway and mitochondria biogenesis, we assessed all these responses in cells under normal gravity in the presence of rapamycin (1g + rapamycin). This approach demonstrated that rapamycin dramatically reduced cell proliferation rates (Fig. 5A) and metastatic activity (Fig. 5B). Interestingly, rapamycin treatment, which inhibited the mTORC1 pathway (Fig. 5C), up-regulated the level of AMPK phosphorylation (Fig. 5C), and induced mitochondria biogenesis in cells under 1 g condition (Fig. 5D,E). In contrast, rapamycin treatment dramatically reduced cell glycolysis metabolism (Fig. 5F). Our data indicate that SMG-induced suppression of cell proliferation and metastasis and activation of the AMPK pathway could potentially be mediated by the SMG-induced inhibition of the mTORC1 pathway.

## Discussion

Previous studies showed that SMG altered cytoskeleton organization in tumor cells^15–17^. However, its molecular mechanism is elusive. In this study, we investigated the effect of SMG on cytoskeleton of BL6-10 cells. We demonstrate that SMG alters cytoskeleton by decreasing stress fibers, lamellipodia and filopodia, which is consistent with our previously published observations^24^. To assess the formation of focal adhesions, we stained cells on chamber slides with antibodies binding focal adhesions-associated proteins, paxillin and vinculin, and analyzed them by fluorescein microscopy. Interestingly, we find that SMG significantly reduces formation of focal adhesions (multi-protein complexes controlling cytoskeleton *via* the FAK/RhoA pathway)^4^, consistent with previous reports^30,35^. Furthermore, we demonstrate that SMG dramatically inhibits FAK and RhoA activity, thus clearly indicating that SMG-induced cytoskeletal alterations are at least in part due to the SMG-triggered inhibition of FAK and RhoA signaling.

The AMPK kinase acts as an intracellular energy sensor, which is a key regulator of mitochondrial biogenesis and functions in this regard to maintain energy homeostasis^36^. mTORC1 acts as another energy sensor in mammalian cells and serves as a central cell-growth regulator by responding to growth factors and nutrient signals. Since AMPK is activated upon various cellular stresses, such as nutrition depletion, hypoxia and heat shock^37–39^, we assessed whether SMG affects the AMPK pathway. We demonstrate that BL6-10 cells upregulates production of pAMPK (T172) and enhances AMPK-regulated ULK1 activity in response to SMG condition, indicating that SMG activates the AMPK-ULK1 pathway. We also show that SMG induces mitochondrial biogenesis in cells under SMG. Interestingly, our assessment of the effect of SMG on mTORC1 demonstrates that SMG reduces levels of pAKT (S473), pS6K (S235) and pELF4E (S209) and inhibits cell glycolysis metabolism in melanoma cells, indicating that SMG inhibits the AKT-mTORC1-S6K-ELF4E pathway. Therefore, our data suggest that SMG activates the AMPK but suppresses the mTORC1 pathway *via* the SMG-induced inhibition of FAK and RhoA signaling molecules.

**Figure 6:**
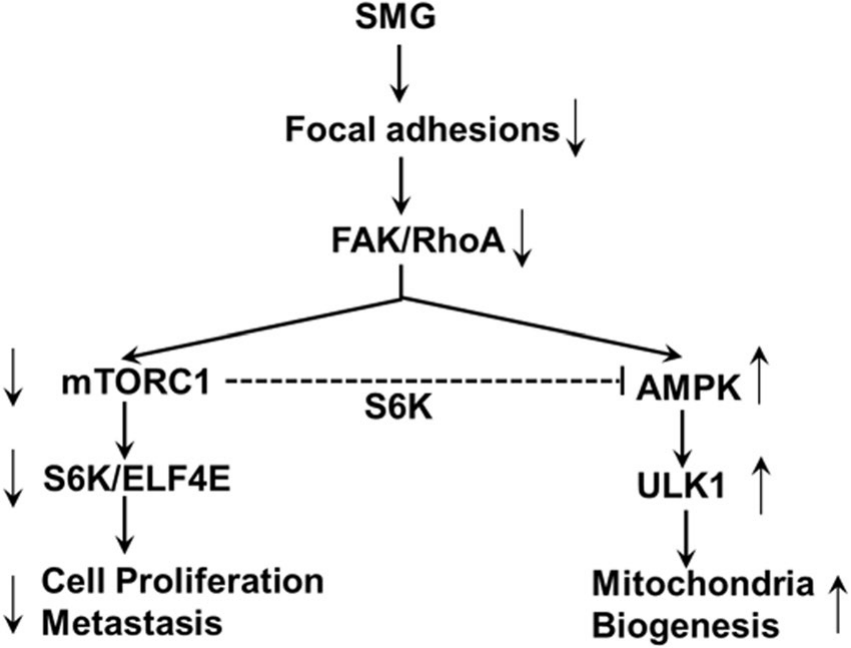
Schematic diagram presenting pathways where SMG-induced inhibition of focal adhesions suppresses FAK/RhoA activation and consequently the mTORC1 signaling and cell glycolysis while activating the AMPK pathway and inducing mitochondria biogenesis, leading to the inhibition of tumor cell proliferation and metastasis.

To further confirm the above finding, we performed the SMG study using CNF1. CNF1 is a broad spectrum activator of Rho family proteins that deamidates and thereby activates RhoA, Rac1 and Cdc42 GTPases^33,34^. It has been reported that CNF1 triggered Rac1-dependent cell invasion^40^. In this study, we demonstrate that CNF-1 toxin activates the upstream signaling (FAK and RhoA) of the mTORC1 pathway and is capable of converting SMG-induced effect on the reduction of cell focal adhesions and inhibition of the mTORC1 pathway and cell glycolysis metabolism. Therefore, we conclude that SMG activates the AMPK but suppresses the mTORC1 pathway most likely through the SMG-induced inhibition of focal adhesions and FAK and RhoA action (Fig. 6).

It has been shown that mTORC1 inhibits AMPK signaling *via* the activation of S6K^41^. We, therefore, assume that SMG-induced activation of the AMPK pathway may occur because of SMG-inhibited S6K within the AKT-mTORC1-S6K-EIF4E pathway, which should result in less S6K-induced inhibition of the AMPK pathway. To assess this assumption, we repeated experiments using an mTORC1-specific inhibitor, rapamycin. Our experiments show that rapamycin efficiently inhibits S6K and ElF4E, suppresses cell glycolysis metabolism, proliferation and metastasis into lungs, while activating the AMPK-ULK1 pathway and inducing mitochondria biogenesis. Taken together, these observations suggest that SMG-induced suppression of the cell proliferation and metastasis and activation of the AMPK pathway are at least in part triggered by SMG-induced inhibition of S6K activity (Fig. 6).

Previous studies showed that SMG inhibited tumor cell proliferation, adhesion and migration^18–20^. However, molecular mechanisms underlying SMG-induced alterations in cell biology have not been identified. Here, we investigated the effect of SMG on the biological characteristics of BL6-10 melanoma cells. We demonstrate that tumor cells aggressively grew under normal gravity using glycolysis, a carbonic metabolism, as a more efficient source to fuel biosynthesis required for fast cellular proliferation. Tumor cell growth is dramatically inhibited under SMG, when tumor cells revert to catabolic metabolic machinery for housekeeping functions, supporting SMG-induced quiescent tumor cells. Tumor aggressiveness is closely associated with tumor metastasis involving multiple steps, such as cell adhesion, migration and invasion^42,43^. MET is a receptor tyrosine kinase for hepatocyte growth factor, that cross-talks to other signaling molecules, leading to regulation of oncogenesis, cell migration and invasion^44^. Integrin α6β4 associates with MET and acts as supplementary docking platform for binding of other transducers to enhance MET signaling^26^. MMP9 is controlled by signaling through FAK and RhoA^45^, and has been found to modulate cell adhesion, migration and invasion^26^ and to support tumor metastasis^27^. We previously demonstrated that cell surface glycoprotein Met72 is associated with high metastasis of BL6-10 cells to lungs^28^. In this investigation, we assessed the effect of SMG on the expression of the above metastasis-related molecules and also on cell proliferation and metastasis. We demonstrate that SMG inhibits expression of integrin α6β4, MMP9 and Met72, leading to significant reduction in cell adhesion and invasiveness *in vitro* and tumor metastasis to lungs *in vivo*. In addition, we also demonstrate that CNF1 is able to convert SMG-induced inhibition of expression of these metastasis-related molecules and SMG-induced alterations in cytoskeleton, focal adhesions, cell proliferation and metastasis. Louis *et al*. previously suggested that small GTPases of the Rho family known to control several aspects of cell dynamics (vesicular transport, traffic and cytoskeleton turnover) might be the key players in mammalian cell adaptation to microgravity^46^. Thiel *et al*. have recently demonstrated that mammalian cells are equipped with a highly efficient adaptation potential to microgravity environment, and indicated that RhoGTPases are interesting candidates to explain the mammalian cell adaptation to microgravity^47^. In this study, we for the first time, reveal that SMG dramatically reduces formation of focal adhesions and inhibits cell proliferation and metastasis through FAK/RhoA-mediated inhibition of the mTORC1 pathway and activation of the AMPK pathway (Fig. 6). Although ground-based simulators of microgravity are valuable tools to study micro-gravitational effect on mammalian cells, they still have their own problems or limitations^48^. Therefore, more explorations have to be performed in the future such as those under the conditions of real microgravity in space to confirm the above observation.

Activation or over-expression of FAK and RhoA in cancer cells has been found to be associated with cancer aggressiveness and metastasis as well as poor patient survival^3,49,50^. Therefore, our observations in this study are consistent with the current FAK/RhoA-targeting cancer therapies that use specific pathway inhibitors^3,49,50^. It was previously demonstrated that SMG inhibits osteogenesis caused by mesenchemal stem cells, but stimulates osteoclastogenesis, leading to bone loss^51–55^. However, molecular mechanisms responsible for these responses are unknown yet. We are currently conducting experiments to assess a hypothetic mechanism, where SMG inhibits formation of focal adhesions of mesenchemal stem cells and osteoblasts, leading to up- and down-regulation of osteoclastogenesis and osteogenesis, respectively, by modulating FAK/RhoA-controlled mTORC1 and AMPK pathways.

Taken together, our observations determine that SMG inhibits focal adhesions, leading to reduced melanoma cell proliferation and metastasis *via* the modulation of the FAK/RhoA-regulated mTORC1 and AMPK pathways. Therefore, our findings may thus have a great impact on our understanding of the effect of SMG on human cell biology and human health.

## Methods

### Ethics statement

All animal experiments were performed in accordance with guidelines and protocols approved by the Animal Use and Care Committee of the University of Saskatchewan (Protocol# 20130020).

### Cells, antibodies and reagents

A highly lung metastatic BL6-10 melanoma cell line was maintained in α-MEM medium with 10% fetal calf serum (FCS)^28^. Rabbit antibodies against ras homolog gene-family member-A (RhoA), ras-related C3 botulinum-toxin substrate-1 (Rac1) were purchased from Santa Cruz Biotechnology (Dallas, TX). Rabbit antibodies against cell division-control protein-42 (Cdc42), focal adhesion kinase (FAK), phosphor-FAK (pFAK, Y397), AKT, phosphor-AKT (pAKT, S473), phosphor-S6K (pS6K, S235) and phosphor-EIF4E (pElF4E, S209), AMPK, phosphor-AMPK (pAMPK, T172), phosphor-ULK1 (pULK1, S375) and integrin α6β4 were obtained from Cell Signaling Technology (Boston, MA). Rabbit antibodies against paxillin and vinculin were obtained from Abcam Inc (Cambridge, MA). Rat anti-Met72 antibody recognizes BL6-10 melanoma cell-surface 72-Kd glycoprotein associated with high tumor metastasis to lung^28^. Monoclonal fluorescein isothiocyanate (FITC)-labeled anti-beta-tubulin antibody and FITC-labeled phalloidin were purchased from Sigma-Aldrich (St. Louis, MO). The cytotoxic necrotizing factor-1 (CNF1), which catalyzes the deamidation of a glutamine residue within the switch-II domain of Rho proteins^56^ leading to activation of Rho proteins RhoA, Rac1 and Cdc42 GTPases^33,34^, was obtained from Dr. Harald Genth, Hannover Medical School, Hannover, Germany^33^. A mTORC inhibitor, rapamycin, was purchased from Selleckchem Inc (Houston, TX).

### Clinostat of simulated microgravity (SMG)

The SM-31 random positional machine (RPM) is a three-dimensional clinostat manufactured by the Center for Space Science and Applied Research, Chinese Academy of Sciences (Beijing, China), which was used to model SMG environment^24,57–59^. The RPM consists of two independent rotating frames, an inner frame and an outer frame. Both frames can rotate randomly at 3-dimension with changes in the acceleration and direction of the samples over time, resulting in randomization of the gravitational vector, low fluid shear stress and three-dimensional spatial freedom. To investigate the gravitational effect, BL6-10 tumor cells were plated into T25 culture flasks or Chamber Culture slides (Nalgene Nunc International Inc, Rochester, NY), and grown for 24 hours to allow cell attachment. The flasks were then filled up with warm culture medium to avoid the presence of any air bubbles, which could lead to shear force-induced damage of cells. The flasks were placed at the center of the inner frame in the RPM, and rotated under simulated microgravity (about 10^–3^ g) at 37 °C in CO_2_ incubator, with 30°/s angular velocity of the rotation. The control cells under ground condition (1 g) were treated as those in the RPM, but placed close to the RPM in the same incubator. Cells were then grown for one to three days at 37 °C in CO_2_ incubator under normal gravity (1 g) or in the clinostat under the SMG condition (µg)^24^. To assess the effect of CNF1 and rapamycin on cells under SMG and 1 g, we applied CNF1 (30 ng/ml)^33,34^ to BL6-10 cells under SMG, and applied rapamycin (5 µM) to BL6-10 cells under 1 g condition, respectively.

### Fluorescent microscopy

For the immunofluorescence staining of microtubules, BL6-10 cells were washed twice with PBS and fixed in 4% paraformaldehyde at room temperature for 15 min. After washing twice with PBS, the cells were permeabilized in PBS containing 0.5% Triton X-100 for 10 min and blocking was done in 1% BSA in PBS at room temperature for 30 min. The cells were incubated with monoclonal anti-beta-tubulin-FITC diluted 1:25 in PBS containing 1% BSA for 1 hour in dark at room temperature. For microfilament fluorescence staining, the permeabilized cells were incubated with FITC-labeled phalloidin diluted 1:20 in PBS for 30 min in dark at room temperature. Propidium iodide (PI, 10 µg/ml) was added 10 min before the ending of the incubation^24^. For measurement of cell focal adhesions, chamber slides were used to grow BL6-10 cells (Nalgene Nunc International Inc), and the permeabilized cells were incubated with anti-paxillin antibody (1:100 diluted in PBS) or anti-vinculin antibody (diluted 1:200 in PBS) containing 1% BSA for 24 hr at 4 °C overnight, followed by the staining with secondary FITC- and PE-labeled anti-rabbit antibody, respectively. After rinsing three times with PBS, plastic chambers were removed, and slides covered with cover slips for fluorescence microscopy^24^. Paxillin and vinculin spots were counted for each cell under fluorescence microscope^30^.

### Western blotting analysis

Cells were harvested and washed twice in ice-cold PBS, then lysed in lysis buffer containing 1% NP40, 0.5% sodium deoxycholate, 0.1% SDS in PBS, supplemented with protease and phosphatase inhibitors, for 30 min on ice with gentle stirring. The lysates were centrifuged and the supernatant was collected. For Western blot, 30–50 μg total protein samples were loaded into each well of the 10% SDS-PAGE gel. After electrophoresis, samples were transferred onto a 0.22 μm polyninylidene fluoride (PVDF) membrane (Millipore, Middlesex County, MA). 5% nonfat milk powder in 1% Tris-buffered saline-tween buffer was used to block membranes. Then, membranes were incubated with required primary antibodies overnight at 4 °C, followed by the incubation with matching horseradish peroxidase-conjugated secondary antibodies. Signals obtained on the membranes with the horse radish peroxidase developer solution were quantified using chemiluminescence. Glyceraldehyde 3-phosphate dehydrogenase (GAPDH) was used as an internal reference.

### *In vitro* tumor cell proliferation assay

BL6-10 tumor cells (1 × 10^5^) were plated into T25 culture flasks, and grown for 24 hr to allow full cell attachment. The flasks were placed at the center of the inner frame, and clinorotated under SMG at 37 °C in CO_2_ incubator after flasks were filled up with culture medium with or without CNF-1 (30 ng/ml)^24^. Cells in flasks without rotation were served as normal gravity (1 g) controls^24^. To assess *in vitro* tumor cell proliferation, cells were harvested daily for three days, and live cell numbers were counted using trypin blue exclusion.

### *In vitro* special assays

To measure biochemical characteristics of analyzed cells, such as NAD(H), glycolysis and mitochondria biogenesis, we performed *in vitro* experiments using NAD+/NADH Quantification kit (BioVision, Milpitas, CA), pH-Xtra^TM^ Glycolysis Assay kit (Luxcell Biosciences, Little Island, Cork, Ireland) and MiltoTracker Green (Life Technologies, Carlsbad, CA), respectively, according to the manufacturers’ manuals^24^. Cellular mitochondria stained with MiltoTracker Green were assessed by flow cytometry and confocal microscopy, respectively^24^. To measure RhoA activity, cell adhesion and invasiveness, we performed *in vitro* experiments using G-LISA RhoA Activation Assay Biochem kit (Cytoskeleton Inc, Denver, CO), CytoSelect™ 24-Well Cell Adhesion Assay kit and CytoSelect™ 24-Well Cell Invasion Assay kit (Cell Biolabs, San Diego, CA), respectively, according to the manufacturers’ manuals.

### *In vivo* tumor cell lung metastasis assay

BL6-10 tumor cells (0.5 × 10^6^ cells/each mouse) were i.v. injected into C57BL/6 mice, and mouse lungs were collected 21 days after tumor cell injection. Black lung metastatic tumor colonies were counted and their nature confirmed by histological examination^28^.

### Statistical analysis

Statistical analysis was conducted using Graphpad Prism-3.0, and statistical significance among groups was analyzed using Student *t* test. A *p*-value <0.05 was considered as significant.
